# Analyzing Infrastructure as Code to Prevent Intra-update Sniping Vulnerabilities

**DOI:** 10.1007/978-3-030-72013-1_6

**Published:** 2021-02-26

**Authors:** Julien Lepiller, Ruzica Piskac, Martin Schäf, Mark Santolucito

**Affiliations:** 8 Eindhoven University of Technology, Eindhoven, The Netherlands; 9 Aalborg University, Aalborg East, Denmark; 10 Yale University, New Haven, USA; 11 Amazon Web Services, NYC, USA; 12 Barnard College, Columbia University, NYC, USA

## Abstract

Infrastructure as Code is a new approach to computing infrastructure management that allows users to leverage tools such as version control, automatic deployments, and program analysis for infrastructure configurations. This approach allows for faster and more homogeneous configuration of a complete infrastructure. Infrastructure as Code languages, such as CloudFormation or TerraForm, use a declarative model so that users only need to describe the desired state of the infrastructure. However, in practice, these languages are not processed atomically. During an upgrade, the infrastructure goes through a series of intermediate states. We identify a security vulnerability that occurs during an upgrade even when the initial and final states of the infrastructure are secure, and we show that those vulnerability are possible in Amazon’s AWS and Google Cloud. We call such attacks intra-update sniping vulnerabilities. In order to mitigate this shortcoming, we present a technique that detects such vulnerabilities and pinpoints the root causes of insecure deployment migrations. We implement this technique in a tool, Häyhä, that uses dataflow graph analysis. We evaluate our tool on a set of open-source CloudFormation templates and find that it is scalable and could be used as part of a deployment workflow.

### 1 Introduction

Managing an infrastructure of thousands of hosts, with different software and servers is nearly impossible to do manually. A relatively new approach to infrastructure management is called *Infrastructure as Code* (IaC). This has given rise to many different tools with a shared goal: helping system administrators manage their infrastructure in the same way as they manage code. Some tools, like Ansible [20], Puppet [23] or Chef [6] are Configuration Management tools: they allow the administrator to specify the entire configuration of one or more running machines and automatically deploy it by connecting to that machine and performing administrative tasks on behalf of the administrator. These tools automatically detect and apply the steps necessary to switch from the current state of a machine to the desired state, specified by the administrator. Similarly, tools like Amazon's CloudFormation [3] or Hashicorp's Terraform [11] read a description of the desired infrastructure and automatically take the necessary

(a) The initial deployment

(b) An insecure update order

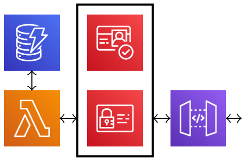

(c) A secure update order

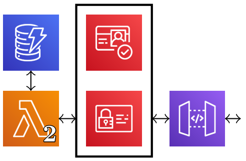

(d) The target updated state

Fig. 1: A deployment of a computation (the orange lambda), accessing a database (the blue disk stack), which is accessible to the outside world through an API (the purple gateway). The upgrade should change the computation to access more sensitive data (the lambda with the subscript 2), but be authenticated through a user check (the red identification checks).

steps to deploy that infrastructure. In CloudFormation, an infrastructure configuration is declared as a set of *resources*.

Benefits of IaC are well-known among practitioners: the entire infrastructure is described accurately by a configuration file, making it easy to debug or visualize the infrastructure. This way the infrastructure can be version controlled and documented as any other programming language. The tools help guarantee identical configuration of hosts, making it an essential practice for security and maintainability.

However, for all the benefits IaC brings, it also opens new security vulnerabilities. We have identified a new class of vulnerability issues that appear while the tool is operating on the infrastructure. In order to decrease infrastructure upgrade times, deployment tools typically will run many operations in parallel. We argue that this parallelism, as well as the global naming used in these infrastructures, can lead to discrepancies during the upgrade that lead to a violation of the intended security policy, *even if* the initial infrastructure *and* the target infrastructure are both perfectly secure. We empirically validate our claims by reenacting this vulnerability in both, Amazon's AWS and in Google Cloud.

#### 1.1 Proof of Concept

When upgrading the infrastructure, if operators do not provide enough dependencies, ie. they do not impose an ordering on upgrade operations, a security policy and a protected service might be upgraded in an order that exposes private data. Consider an example given in Figure 1: an API service that replies to any request with some benign information, as depicted in Fig. 1a. The service is upgraded so that the API returns private information about users, and the security policy is modified to allow only authenticated users to access the service, as shown in Figure 1d. This architecture is a core architectural building block for serverless computing. This same configuration is recommended in AWS's "Well Architected" developer guideline series [1]. The upgrade code is functionally correct and implements the desired change, but the user did not specify ordering constraints. However, without such constraints, there are two possible upgrade plans. First, as shown in Figure 1b, the backend computation may be updated first. In this case, since the authentication has not yet been added to the API, there is a short period of time where private data is publicly accessible. The amount of time this information is exposed depends on the cloud service provider and the particulars of the infrastructure, but typically ranges on the order of seconds to minutes. We call this kind of attack intra-update sniping vulnerability. The second possible upgrade order, shown in Figure 1c, implements the desired secure update order. Enforcing the second ordering requires the user to explicitly specify an ordering constraint that the authentication must be added before the backend computation is updated.

Another instance of intra-update sniping vulnerability happens when components are added or removed from an infrastructure, but no ordering constraints are given between them and components that use them. As an example, suppose a user is adding a lamda that reads data from a new S3 bucket. If no dependency is specified, the lambda could be created and connected to the bucket before CloudFormation recognizes that the bucket is already owned. The attacker who owns this bucket may then inject their data into the user's system during the time it takes CloudFormation to notice the naming conflict and roll back the migration. This is related to the issue of S3 bucket namesquatting [15].

Although this paper is mostly focused on Amazon's infrastructure, we have successfully reproduced a similar scenario in Google Cloud, demonstrating that intra-update sniping vulnerabilities are not limited to one cloud provider. We reported this issue to Google, and although they acknowledged the problem, they explicitly stated that it is the responsibility of the user to ensure the security of their deployment.

#### 1.2 Detecting Intra-update Sniping Vulnerabilities

We propose a tool, Häyhä, that detects possible intra-update sniping vulnerabilities and proposes solutions to users. Häyhä allows CloudFormation users to check the security of planned updates to their infrastructure, before they actually deploy the update. Although our tool is specifically engineered to work with CloudFormation, this class of vulnerabilities is not limited to it, and the proposed solution is generic enough to be adopted in any other Infrastructure as Code language.

The main challenge in detecting intra-update sniping vulnerabilities is in determining the underlying issue with common deployment models that lead to the security vulnerability. We identify parallelism and in-place upgrades as the root causes, arguing there is a trade-off in Infrastructure as Code between security and scalability. On the opposite side of this trade-off, some practitioners advocate for Immutable Infrastructure [12] management, which re-builds the entire infrastructures from scratch on each update and only switches atomically to the new infrastructure when it is ready. This practice would guarantee atomicity of updates to the infrastructure and the absence of intra-update sniping vulnerabilities. However, this comes with a huge cost in terms of scalability and does not apply well when statefulness is required (for example, migrating an existing database), making it a less attractive practice.

Naturally, there is a connection between intra-update sniping vulnerability and the problem of data races and concurrent access. Our proposed solution, of adding ordering constraints, is somewhat similar to generic tools in the concurrency domain, such as memory barriers or locks [19,16,24], that add constraints to the order of execution of a program. However, the focus of our work are configuration files that describe infrastructure, not programs. We cannot simply apply existing work, because these configuration files do not have a formal semantics, creating this way an additional challenge for our problem domain.

In summary, we identify the following key contributions of this paper:

- The description of intra-update sniping vulnerabilities and how they arise in IaC services, with examples in AWS and Google Cloud.
- An intermediate representation of IaC configurations that allows us to reason about security and network properties of a deployment, as well as about changes in deployments.
- A tool, Häyhä [17] that statically checks for potential intra-update sniping vulnerabilities in a proposed infrastructure update.
- An evaluation on CloudFormation files scraped from GitHub, showing Häyhä scales and runs fast enough to be adopted into developer workflows.

### 2 A Model for Infrastructure as Code

Our tool, Häyhä, detects the possibility of a sniping attack in future deployments. It analyzes the given deployment and raises alarms when it detects potential security issues. The tool follows steps that we further detail in this section.

**Step 1: Internal representation.** First, Häyhä reads the configuration of the current and target infrastructure and translates them to the internal representation. This representation is a dataflow graph identifying which component of the infrastructure has access to which other components, and under which security assumptions. Figure 2 shows two such simplified dataflow graphs that our tool built from arch in Fig. 1. From this graph, Häyhä learns the desired security level of each component. In this section we describe how to compute security levels of resources in a given CloudFormation file: in Section 2.1 we describe the concrete syntax of a general CloudFormation file and how it applies

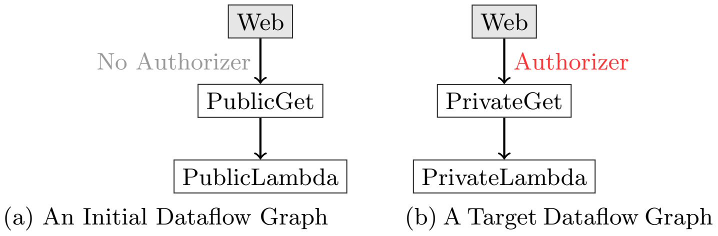

Fig. 2: Dataflow graphs derived from an infrastructure to other IaC tools; in Section 2.2 we describe how we model an infrastructure in terms of network communication and security; finally, in Section 2.3 we show the execution semantics and computation of the security level of resources in an infrastructure.

**Step 2: Capturing all potential upgrade states.** After the initial and target configurations are converted to our model, Häyhä builds an *upgrade state*, designed to represent every possible intermediate infrastructure that could exist during the upgrade. In Section 2.4 we formally define the upgrade semantics from an initial state to a target state in terms of our model, while in Section 3.1 we show how the upgrade state is built in practice. Figure 3 shows such a state, in form of a graph, which contains a path (*Web* to *PublicGet* to *PrivateLambda*) allowing any user on the web to access a sensitive resource in a non-secure manner. Finally, in Section 3.2 we discuss how dependency relations refine the upgrade state.

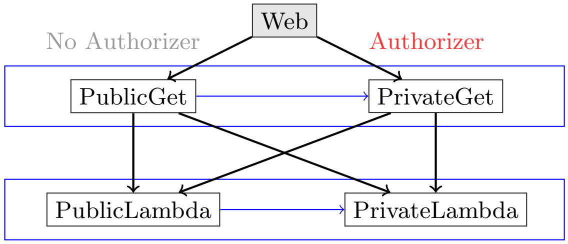

Fig. 3: Upgrade State with a Path Exposing a Security Vulnerability

**Step 3: Analysis.** (Section 3.3) Häyhä computes an over-approximation of the intermediate states and the security level of their nodes in order to answer two questions: 1) is every node in every possible intermediate state at least as secure as the corresponding node in the initial or target configuration? and 2) does every node in every possible intermediate state communicate only with existing nodes? Any possible violation is reported to the user so they can take action and modify their target configuration accordingly. For example, using the

`DependsOn` keyword, one can enforce build orders in a CloudFormation file. For Figure 3, Häyhä reports the possible insecure access to PrivateLambda:

`Resource PrivateLambda is not sufficiently protected, it needs at least Authorizer and is protected by None during upgrade. Add DependsOn properties to ensure correct security.`

### 2.1 CloudFormation Infrastructures

CloudFormation uses a declarative language in which users can specify the desired state of their system. An example of a CloudFormation file is given on the left side of Figure 4. It shows a simplified example of an infrastructure in which an API can be called to access the result of running a Lambda (a simple function). There are no formal semantics for CloudFormation files [4,9] – they are simply YAML or JSON files created from the given AWS CloudFormation templates. Other tools, such as Terraform by HashiCorp, follow a similar template-based design.

To formalize the behavior of IaC languages, we would also need to formalize the precise behavior of components. However, these components are very diverse, ranging from firewalls and HTTP servers to general purpose machines or even entire network configurations. Fortunately, the intra-update sniping vulnerability is independent from the precise behavior of individual components, and we only need to analyze the network and security behavior of the infrastructure. We only track the security level of requests, and abstract away from their content. To describe our model, we need to introduce three concepts used in IaC:

A component of the infrastructure is called a *resource*. Every configuration file declares a set of resources and their configurations (e.g. Figure 4). Some resources, like the *LambdaExecutionRole* and the *LambdaPermission* are security resources, and they prevent an unauthorized use of other resources. Other resources, like the *GreetingLambda* and the *GreetingRequestGET* are actual running processes, the later also being publicly accessible. Finally, some resources do not correspond to a running process, but to a group of resources such as *GreetingApi* that gives some configuration value to every resource in the group.

A resource's configuration may reference other resources, and we record that information in our model. Based on the CloudFormation documentation, we distinguish different types of references that we list below:

– **network references(r, r')** are directed network connections between two components $r$ and $r'$, that allow $r$ to send requests to $r'$, and receive answers.
– **incoming protection references(r, s)** protect all incoming requests to a resource $r$, using a security resource $s$.
– **outgoing protection references(r, s)** protect all outgoing requests from a resource $r$, using a security resource $s$.
– **connection protection references(r, r', s)** protect a specific connection between two resources $r$ and $r'$ using a security resource $s$.
– **collection references(c, r)** specify a resource $r$ is in a specific collection resource $c$.

| CloudFormation File | Corresponding Model |
|---|---|
| { ”Resources”: {<br>”LambdaPermission”: {<br>”Type”: ”AWS::Lambda::Permission”,<br>”Properties”: {<br>”FunctionName”: ”GreetingLambda”,<br>”SourceArn”: ”GreetingApi”<br>}<br>}, | LambdaPermission [*security*]<br>intrinsic security: LambdaPermission,<br><br>connection security(GreetingApi, GreetingLambda, *this*) |
| ”GreetingLambda”: {<br>”Type”: ”AWS::Lambda::Function”,<br>”Properties”: {<br>”Role”: ”LambdaExecutionRole”<br>}<br>}, | GreetingLambda<br>intrinsic security: ⊤ |
| ”GreetingRequestGET”: {<br>”Type”: ”AWS::ApiGateway::Method”,<br>”Properties”: {<br>”Integration”: ”GreetingLambda”,<br>”RestApiId”: ”GreetingApi”<br>}<br>}, | GreetingRequestGET [*public*]<br>intrinsic security: ⊤,<br><br>network(*this*, GreetingLambda),<br>collects(GreetingApi, *this*) |
| ”GreetingApi”: {<br>”Type”: ”AWS::ApiGateway::Api”<br>}, | GreetingApi [*collection*]<br>intrinsic security: ⊤ |
| ”LambdaExecutionRole”: {<br>”Type”: ”AWS::IAM::Role”<br>”Properties”: {<br>...<br>}}}} | |

Fig. 4: Mapping Between a CloudFormation File and our Model

Each of these reference types can be present in any resource, any number of time. The resource it is declared in can take any role in the relation that it defines, and we represent the resource as *this* in the model, as shown on the right side of Figure 4.

In CloudFormation, a dependency is declared by using e.g. the *DependsOn* keyword. A dependency restricts the order in which updates can occur: before a resource can be updated, all the resources it depends on must have been updated.

### 2.2 Model of a CloudFormation Infrastructure

We now describe a model for a CloudFormation infrastructure. We define a *state* $S = (R, D)$ as a set of resources and a partial order that represents the dependency relation between resources. A resource is a tuple composed of a name (string), a type, an intrinsic security context, an origin flag, the different types of references discussed above, and the original configuration of the resource.

With $(id, id') \in D$ we denote that $id$ depends on $id'$, and that $id$ cannot be upgraded until $id'$ is upgraded.

The origin flag denotes whether the resource comes from the initial state or the target state during an upgrade, but it is not used at all when dealing with a single state. Similarly, the original configuration's type is not further defined, and depends on the vendor. It is not used for a single deployment, and we only use it to check for equality of resources when updating an existing deployment.

Inspired by Abstract Interpretation [10], we define a security context as an abstract domain with a partial order and some abstract operations: a top, a bottom, a meet, and a join. When two security contexts are comparable ($x \sqsubseteq y$), we say that $x$ is less permissive than $y$, or that $x$ is more secure than $y$.

We define predicates that can help us to express some properties of resources in a specific state $S$: *collection*$(r)$, resp. *security*$(r)$, means that $r$ is a resource whose type is that of a collection resource, resp. a security resource. We use *public*$(r)$ to denote when $r$ is a resource whose type is that of a resource that can be accessed from anywhere on the internet (although this might be restricted with security references), or if it is contained in a collection that is itself publicly accessible.

**Definition 1 (connection).** *A connection is possible between two resources when there is a network reference between them or resources that collects them.*

$$ref(r, r') \iff \exists c, c'. \wedge \begin{cases} network\ reference(c, c') \\ r = c \vee collects(c, r) \\ r' = c' \vee collects(c', r') \end{cases}$$

The security of a connection is the minimum security level a request from $r$ must have to be able to reach $r'$ directly. This definition reflects the fact that, when a connection is secured by multiple security resources, it must have sufficient authority to be accepted by *all* of them.

**Definition 2 (connection security).**

$$security(r, r') \iff \sqcap \left\{ sec(s) \;\middle|\; \begin{array}{c} \exists c, c'. \vee \begin{cases} incoming\ protection(c, s) \\ outgoing\ protection(c', s) \\ connection\ protection(c, c', s) \end{cases} \\ with\ \wedge \begin{cases} (r = c \vee collects(c, r)) \\ (r' = c' \vee collects(c', r')) \end{cases} \end{array} \right\}$$

#### 2.3 Execution Semantics

The execution semantics for our intermediate representation is given below. The semantics explains which resources are allowed to talk to which resources, and under which security level. When we write $L \vdash r \rightarrow r'$, it means that $r$ is allowed to send a request to $r'$, under the security level $L$.

A request can come from the internet (represented with the constant $W$) and reach a public resource $r'$ if it has a sufficient security level $L$. Similarly, a request can come from a resource $r$ and reach $r'$ if it has a sufficient security level, $r'$ is not a collection, and both resources have an adequate configuration that allows them to communicate.

$$\text{OutsideRequest}\ \frac{r' \in R \quad \neg collection(r') \quad L \sqsubseteq security(W, r') \quad public(r')}{L \vdash W \rightarrow r'}$$

$$\text{InternalRequest}\frac{(r, r') \in R^2 \quad \neg collection(r') \quad L \sqsubseteq security(r, r') \quad ref(r, r')}{L \vdash r \rightarrow r'}$$

A path $P$ is a finite sequence of resources whose first resource is public, and subsequent resources can be reached from the previous, using the above semantics under some security level. The security of a path is then defined as the minimal security level under which every node can be reached in the above semantics:

$$security((r_1, \ldots, r_n)) = \wedge_{i=1}^{n} security(r_{i-1}, r_i)$$

with $r_0 = W$. We note $W \rightarrow^* r$ the set of paths whose last element is $r$. Similarly, the security of a node is defined as the minimal security level under which the node can be reached by at least one path:

$$Sec(r) = \vee \{security(P) | P \in W \rightarrow^* r\}$$

When the infrastructure, under which we consider the security of resources, is not clear from the context, we clarify that with a subscript $Sec_S(r)$.

**Definition 3 (Substate).** *When comparing two states, $S_1$ and $S_2$, we say that $S_1 \subseteq S_2$ when*

- *Every resource of $S_1$ is a resource of $S_2$ and*
- *For every pair of resources $r, r'$ in $S_1$, if $L \vdash r \rightarrow r'$ holds in $S_1$, then it also holds in $S_2$.*

Our first lemma states that, when a state is a substate of another, its nodes are at least as secure as the other.

**Lemma 1 (Substate Security).**
$\forall S_1, S_2.\ \forall id \in S_1.\ S_1 \subseteq S_2 \implies Sec_{S_1}(id) \sqsubseteq Sec_{S_2}(id)$

*Proof.* We note that by definition, *id* is in both states. Additionally, any path in $S_1$ is also a path in $S_2$, and since the security of connections in $S_1$ is more secure than the same connections in $S_2$, the security of paths in $S_1$ is greater than the security of the same paths in $S_2$.

The security of a node is the meet of the security of paths that lead to it in the state. Paths that lead to *id* is $S_2$ are the paths that lead to it in $S_1$, and potentially additional paths. Therefore, the security of *id* in $S_1$ is greater than in $S_2$.

### 2.4 Upgrade Semantics and Security Policy

In IaC tools, an upgrade changes a given infrastructure state to a new state. This is done by upgrading each node that needs to be changed as specified by the new configuration. Generally, nodes are upgraded in an unspecified order, even in parallel, to improve deployment speed. Node updates are sent asynchronously to every service that needs to be updated, and there are dozens if not hundreds of steps each service must take to complete its update. When these upgrades are sent in parallel, it is difficult to reason about the state of the system as the running time for a node upgrade depends on the latency of the service. To model this behavior, we define an interleaving semantics for upgrades.

An upgrade starts in an initial state $S_i$ and ends in a target state $S_t$. Additional dependency ordering information is provided by the relation $D$ of the target state.

The configuration of an identifier can be updated if all its dependencies are already updated ($\forall id', (id, id') \in R \implies S(id') = S_t(id')$), and it has not been updated yet:

$$\text{UpgradeConf}\frac{S(id) \neq S_t(id) \quad \forall id', (id, id') \in R \implies S(id') = S_t(id')}{S \to S[id \leftarrow S_t(id)]}$$

A new resource can be created under the same conditions, if it was not present in the initial state:

$$\text{UpgradeAdd}\frac{id \notin S \quad \forall id', R(id, id') \implies S(id') = S_t(id')}{S \to S[id \leftarrow S_t(id)]}$$

An identifier can be removed, if it is not in the target state:

$$\text{UpgradeDel}\frac{id \notin S_t \quad id \in S}{S \to S \setminus id}$$

We collect every accessible intermediate state in a set denoted by *Acc*:

$$\text{AccInit}\frac{}{S_i \in Acc} \qquad \text{AccNext}\frac{S \in Acc \quad S \to S'}{S' \in Acc}$$

Note that, in the absence of any dependency, Acc contains every combination where each resource is either at its initial or target configuration, leading to $2^n$ possible intermediate states when $n$ is the number of changed resources.

We next show that, when two identifiers are in a dependency relation, some intermediate states are not possible. For ease of expressing this lemma, we extend equality to also check whether $id$ is in the domain of $S$. If $id$ is neither in $S$ nor $S'$, we have $S(id) = S'(id)$. Otherwise, $id$ must be in both and associated to the same configuration for the equality to hold.

**Lemma 2 (Dependency Restriction).**
$\forall (id, id') \in R, S \in Acc \implies S(id) \neq S_t(id) \vee S(id') \neq S_i(id') \vee S_t(id) = S_i(id) \vee S_t(id') = S_i(id')$

*Proof.* By induction of $S \in Acc$ and by case analysis on the inequality that holds in the inductive case.

We now define the security policy as:

**Definition 4 (Security Policy).** *A deployment from $S_i$ to $S_t$ is secure iff:*

$$\forall S \in Acc, \forall id, \begin{cases} Sec(S, id) \sqsubseteq Sec(S_i, id) & \textit{if } S_i(id) = S(id) \\ Sec(S, id) \sqsubseteq Sec(S_t, id) & \textit{if } S_t(id) = S(id) \\ Sec(S, id) = \bot & \textit{otherwise (id is not in S)} \end{cases}$$

Our work focuses on security issues that happen during upgrades, assuming that the initial and target states are both secure. We require that in any intermediate state any resource is at least as secure as their counterpart in the initial or target state, depending on where their configuration comes from.

## 3 Architectural Design of the Häyhä Tool

### 3.1 Upgrade States

To verify the security of intermediate states, we could compute all the possible intermediate states and pass them to existing tools that could check the security of such states. However, this approach has two main drawbacks. First, we would need to construct $2^n$ intermediate states, which does not scale for large infrastructure changes. Second, the result of such tools would not be easy to understand for end users, as they would report issues with states that are not defined or even considered by the user. Our goal is a tool that is both scalable and able to provide suggestions on how to change the target configuration, not some hidden intermediate configuration.

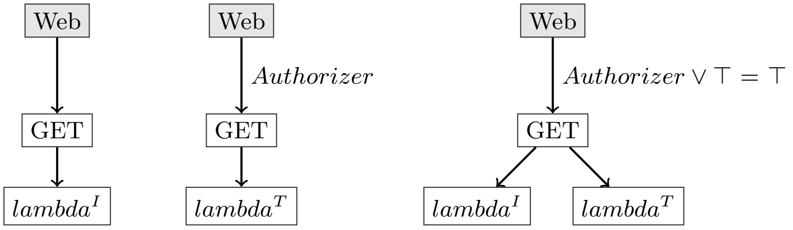

(a) Graphical Representation of the Initial State (b) Graphical Representation of the Target State (c) Graphical Representation of the Upgrade State

Fig. 5: Example Upgrade State

To address scalability we introduce *upgrade states* which represent multiple states on which we can apply the same execution semantics. Recall that a state is composed of a list of resources with their origin, type and references, and of a dependency relation. An upgrade state is composed in the same way. The set of resources is the union of the resources from the initial and target states, excluding initial resources that only differ from their target counterpart by their provenance flag. When resources are added or removed from an infrastructure, we introduce an empty resource for each of them. They represent the absence of these resources. The dependency relation of the upgrade state is the dependency relation of the target state.

The execution semantics of an upgrade state is the same as the execution semantics of a normal state. Since the upgrade state represents multiple versions of the same resources at the same time, we need to change the definition of the security level of a connection between resources. An example of an upgrade state is given in Figure 5. The initial state has an API, a GET method and a lambda, and everything is public. The target state modifies the lambda and adds an authorizer. The upgrade state is comprised of the unchanged API, the target authorizer (with an empty resource as its initial counterpart), the GET method (which did not change), and the two variants of the lambda. The connection to the GET method is protected either by the empty node ($\top$) or the target authorizer. The minimal security level for this connection is therefore $\top$.

In summary, when a security resource is relevant for a connection, we need to consider its counterpart that has a different provenance flag. If it is also relevant, the connection is protected by the disjunction of the security level of these resources (they cannot both exist at the same time, but one of them exists at any given time). If it is not relevant, the upgrade state represents at least one case where the security resource is not relevant, meaning that the connection is protected by the disjunction of the first security level and $\top$, which is $\top$ (no security at all). If the counterpart is an empty resource, the upgrade state represents at least one case where the security resource was deleted (or not yet added), so the connection is also unprotected. If there is no counterpart, the connection is simply protected by the resource, because it does not change in any way during the upgrade.

We denote by $U(S_i, S_t)$ the upgrade state created from the initial state $S_i$ and the target state $S_t$. We now show that this state indeed collects all possible intermediate states.

**Lemma 3 (Upgrade Graph is an Overapproximation).**
$\forall S \in Acc. S \subseteq U(S_i, S_t)$

*Proof.* To apply the definition, we first show resources of $S$ are resources of $U$. Then, we show that any connection in $S$ is a connection in $U$, because resources come with the same references in both states.

#### 3.2 Splitting Dependencies

We have seen that the upgrade state created from the initial and target configurations is an over-approximation of all the intermediate states, when we do not consider dependencies. Because dependencies reduce the number of intermediate states, the upgrade state might not be precise enough and might produce a warning when no actual intermediate states violate the security policy.

**Variants**. When the state has two nodes $A$ and $A'$ with the same identifier, but a different label, we call them a variant of one another. When $A$ belongs to the initial configuration and $A'$ to the target configuration, $(A, A')$ is called an upgrade pair.

We refine the upgrade state by splitting it along a dependency. Considering a state $S$, its dependency relation $D$, and two target resources $(A', B') \in D$, the split of $S$, $split(S, A', B')$ is a set of upgrade states. Suppose $A'$ and $B'$ are, respectively, part of an upgrade pair $(A, A')$ and $(B, B')$. Then, $split(S, A', B')$ is the set of three upgrade states, where only one of $A$ or $A'$ remains, and only one of $B$ or $B'$. We exclude the case where $A'$ and $B$ remain. When any of these nodes does not exist, the number of possible combination is reduced. When only $A'$ and $B$ exist in $S$, we have found an impossible situation, and the result of splitting is the empty set.

Although this process creates an exponential number of states, the number of dependencies tends to be limited in practice, because they slow upgrades down. At the same time, a big number of dependencies actually reduces the number of possible intermediate states, until every node is in a dependency, in which case there are exactly $n$ intermediate states.

We now prove that splitting the upgrade state is correct, in the sense that the set of states $split(S)$ still contains all the possible intermediate states ($Acc$):

**Theorem 1 (Correct Split).**
$\forall S \in Acc.\ \exists u \in split(U(S_i, S_t)).\ S \subseteq u$

*Proof.* Let us take a state $S \in Acc$ from the set of all possible intermediate states. Since splitting a state according to a dependency preserves the states from $Acc$ (Lemma 4 below), we can consider every dependency and split them in any order. Initially, it holds that $S \subseteq U(S_i, S_t)$, using Lemma 3.

Consider an upgrade state $u$ such that $S \subseteq u$ and $D(id, id')$. By Lemma 4, we can find a state $u' \in split(u, id, id')$ such that $S \subseteq u'$.

After applying this for each dependency, $u'$ is one of the states resulting from $split(U(S_i, S_t))$, and the claim of the theorem holds.

The following intermediate lemma is needed to prove the correction of the split. It states that if a state contains one of the accessible states, splitting a dependency in it results in a set of states, where one of them still contains this intermediate state.

**Lemma 4 (Split Graphs).** $\forall S \in Acc.\ \forall (id, id') \in D.\ S \subseteq u \implies \exists u' \in split(u, id, id'), S \subseteq u'$

*Proof.* Take $(A, A')$ the upgrade pair whose identifier is $id$. Similarly, take $(B, B')$ the upgrade pair whose identifier is $id'$. Since $S \in Acc$, $A'$ and $B$ cannot both exist at the same time in $S$ (Lemma 2). Since $S \subseteq u$, we also know that $u$ has at least one variant of $id$ and one variant of $id'$, the ones that are present in $S$.

The states from $split(u, id, id')$ are composed of the same nodes as $u$, except for $id$ and $id'$, where they all have one of the four possible combinations of initial and target states, except for the pair $A', B$. Since $S$ doesn't have them both either, one states has the same variants of $id$ and $id'$ as $S$, and we call it $u'$. We now show that $S \subseteq u'$.

First, we note that $u'$ has the same nodes as $u$, except for those with identifier $id$ and $id'$. For any resource in S, the resource was present in $u$, so it is also in $u'$, unless it has identifier $id$ or $id'$. For this last cases, we note that $u'$ is defined to contain the same variants as $S$, so the resources of $S$ are also resources of $u'$.

Second, if we take $L \vdash r \rightarrow r'$ in $S$, we can use the same reasoning as in Lemma 3 to conclude that is also holds in $u'$. Thus we conclude that $S \subseteq u'$.

#### 3.3 Finding Vulnerabilities

After Häyhä constructs the upgrade state, the next step is to check for security issues. Although we could split the upgrade state recursively until no dependency remains, a more interesting strategy is to immediately check the upgrade state for issues. If none is found, it is not necessary to refine the upgrade state. Otherwise, we try to find a relevant dependency and split the upgrade state on it, running the analysis on the resulting states, splitting on other dependencies as needed.

Our analysis detects two types of issues: first, if an empty node is accessible, it might be used by the infrastructure at a point it is not registered by the owner of the infrastructure. This is the case for a new node that is accessible before it is created. When that node is a resource that can be claimed by a third party (such as an S3 bucket), the attacker might be able to register it before the user. Similarly, for a deleted resource, an attacker could register it for themselves before the user stops using it.

Second, the security context of every node in the upgrade state is compared to the security of the same node in the initial or target state (depending on its provenance flag). When its security is strictly lower than the security of the node in the state it comes from, or incomparable, we raise an alarm because there is an intermediate step where the resource might not be sufficiently protected.

Using Lemma 1 and Theorem 1, when the security of a node in a possible intermediate state (collected in $Acc$) is insufficient, the security of that node in at least one split upgrade state is even lower. Therefore, if there is a violation of the security property, our tool will detect it.

### 4 Experiments

Häyhä is designed to be used before the deployment of a CloudFormation update, and it is crucial that Häyhä does not interrupt developer workflow. Our goal was, therefore, to evaluate the scalability of Häyhä on a variety of real-world CloudFormation updates. To do this, we collected 36 CloudFormation files from GitHub, where each file had a history of updates (commits). We ran Häyhä against every update recorded in GitHub to that file, and measured the running time. We found that our analysis completed within one seconds for all files – we believe that these results indicate that Häyhä could be integrated in developer workflow with minimal disruption to the user. The details of the evaluation dataset are given in Fig. 6.

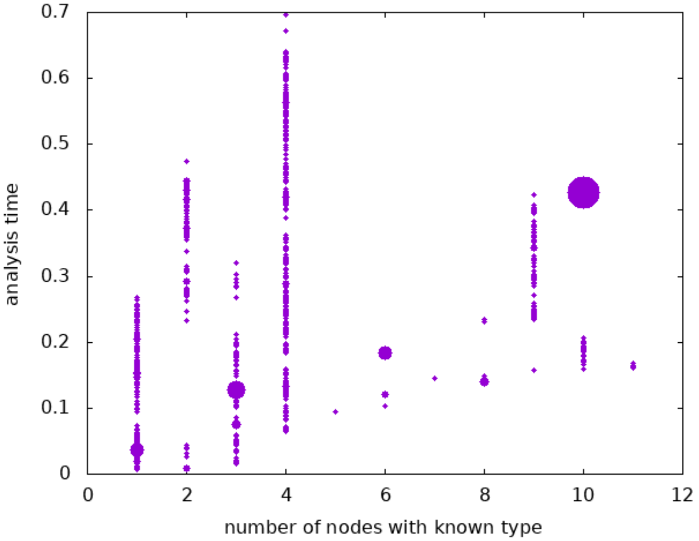

Fig. 6: Analysis time of various CloudFormation files from GitHub. Point size is proportional to the number of updated resources, which are between 0 and 31.

To collect the set of GitHub CloudFormation files used in our scalability benchmark, we searched GitHub using the web search tool for code with the keyword `AWSTemplateFormatVersion` - which is a required keyword for any CloudFormation file. We then filtered by the `.yaml` extension, and further manually filtered for valid CloudFormation files (as opposed to other languages with overlap). Since we wanted to track updates to these files, we also filtered manually to find only files that had a revision history ($\geq 2$ commits for the file).

While we showed that Häyhä scales well on real world data, we did not identify any instances of intra-update sniping vulnerability in these files. This is an expected result, as the CloudFormation files we found on GitHub were generally designed as templates that developers would customize to their own needs. We believe application-focused CloudFormation files are not often uploaded, since CloudFormation files can contain sensitive and proprietary information (e.g. infrastrucuture design). In order to run a large-scale analysis to check for past instances of intra-update sniping vulnerability, we would need access to a repository of the private user data for many CloudFormation users.

### 5 Related Work

Following the development and use of Infrastructure as Code (IaC) practices, many threats and security challenges were recognized [26,27]. The security risks that have been identified in IaC have thus far remained similar to existing vulnerabilities arising from poor security practices, such as infrequent key rotation and hard-coded secret values [25]. Additionally, despite existing recommendations and good practices when dealing with cloud infrastructure, many existing deployments are still left insecure by user misconfigurations. For example, storage "buckets" which host files, should generally be configured by user to disallow world readable/writable permissions. However, in practice, users struggle with this [8]. Existing work has used SMT solver to automatically detect such vulnerabilities and help users secure their resources [4,9]. In contrast, we focus on the dynamic behavior of deployment updates that occur when using IaC tools, and their effect on security configuration.

Much work has focused on the security of virtualization technologies based on attack models such as malicious cloud users to compromised cloud providers, as summarized in [13]. In our work however, we do not make any assumption on the specific technology, as intra-update sniping vulnerabilities rely mostly on timing and insecure configuration on the user's side.

Our work is based on a graph model of the dataflow network of resources created in an infrastructure configuration. Similarly, Al-Shaer et al [2] propose to model and check network security using a graph-based model of the network. As with other work on the network and infrastructure security [5,18], the focus of the analysis is on the security of static network topologies, instead of the security of a moving topology, as we have in this paper. The analysis of security in static networks and static information flow models [21] is complementary to our work, as we assume the initial and target infrastructure are secure.

Beyond network configurations, there has been work in the analysis of configuration files. In particular, static analysis has been used to check that IaC configurations are idempotent [14,30], an important property for maintaining reproducibility of infrastructure. The reproducibility of infrastructure is known to be a challenge [7], despite IaC being declarative and version controlled. Further efforts have used probabilistic modelling to learn constraints on configurations [22,28,29].

### 6 Conclusion

We have identified a new class of vulnerability that applies to Infrastructure as Code services, intra-update sniping vulnerabilities, that arise from a lack of ordering in upgrading resources. We presented a tool, Häyhä, that detects such vulnerabilities in CloudFormation, and gives feedback to users on how securely update their infrastructure deployment. Our evaluation shows the scalability of Häyhä by running it on existing configurations from GitHub and found that it runs quickly enough to be usable in practice.

### Acknowledgement

This work was completed while working on the grant supported by the National Science Foundation under Grant No. CCF-1715387, and partially supported by the Office of Naval Research under Grant N00014-17-1-2787.
